# Ligature of the Common Femoral Artery; or of the Femoral between Poupart’s Ligament and the Profunda

**Published:** 1854-10

**Authors:** Frank H. Hamilton


ART. II.—*Ligature of the Common Femoral Artery; or of the Femoral between Poupart's Ligament and the Profunda.* By FRANK H. HAMILTON, M. D.

"Some surgeons have doubted the propriety of tying the artery between the going off of the profunda and the origin of the epigastric. We have, however, several times put a ligature here, and in every instance with success."—*Mott.*

Mr. Fergusson would rather tie the external iliac. I have no time to examine the statistics of this rare operation, nor indeed to determine its relative value as compared with the operation of tying the external iliac; but I propose simply to record the case in which I have recently operated. It is known that the objections to this operation consist chiefly in the danger of secondary hemorrhage, from the necessity of tying so close below the pudendal, circumflex and epigastric arteries; and in the difficulty of the operation from the presence of the inguinal glands and their arteries, together with the arteries just mentioned.

With Mr. Skey, such secondary hemorrhage did not occur, in a case which he has related; nor did it occur in my own case. It is in this view that I understand Dr. Mott to call his operations *successful*. I regret that he has not been more particular, but I suppose he would call any operation of deligation successful in which secondary hemorrhage did not occur, and the patient recovered. Although, therefore, my patient ultimately lost his leg, yet I may speak of the deligation as a successful operation; and it is plain that if ligating the artery below Poupart's ligament may cause the death of the limb, it is even more probable that ligation above will produce the same result. So far as the question of arterial supply, after the operation, is concerned, the argument is wholly in favor of this point; if, therefore, by a sufficient number of cases, it can be shown to be as safe against secondary hemorrhage as ligature of the external iliac, then my operation must be preferred. In the hope of aiding in the solution of this question, my dear doctor, I send you this report.

Nov. 10th, 1853. Henry Taintor, æt. —, accidentally shot himself with a Colt's revolver — five barrels being consecutively discharged. One bullet entered his left hand, and one passed through his left thigh. The wound in his thigh was from behind forward, and in a direction which crossed the course of the femoral artery about three inches below the formation of the profunda.

The wounds healed slowly, owing to his exceedingly reduced condition.

On Sunday, Jan. 29, 1854, I was called for the first time to see him. The wound in his thigh had not yet closed, and it was now discharging blood rapidly from an aneurism which had burst into its channel. The aneurismal tumor was very large, extending nearly as high as Poupart's ligament.

Assisted by Drs. Boardman and Pupikofer, I immediately proceeded to tie the femoral artery near where it escapes under Poupart's ligament. The patient was under the complete influence of chloroform.

As soon as the artery was tied the bleeding ceased, and the tumor perceptibly diminished in size.

During the afternoon of this day he was delirious and raving: a circumstance which, I suspect, ought to be attributed to the chloroform. He had, also, retention of urine for twenty-four hours.

Jan. 30. No sensibility below the knee; violent pains in knee, extending into leg; muscular spasms in the leg. On the outside of about the middle of his leg, appeared a white circular spot, of the size of a 25 cent piece, and which resembled an old scar. It was more completely insensible than other portions of the leg about it. It did not recognize the prick of a pin. Was white, and skin in other parts mottled. The pulsations of the posterior tibial artery could be faintly felt.

The white spot gradually extended after this date, the toes grew dark-colored and finally black and dry; the pulsations of the posterior tibial artery ceased; the whole limb became œdematous; the death extended almost to the knee, in portions moist and with perfect lines of separation, and in portions dry and attached. During all of this progress the patient suffered intense pains in different portions of his leg, but always most acute about his knee. There was, however, at this point, no inflammation or sign of injury. The same pain existed in the knee prior to the operation, and I believe it to have been occasioned by some injury inflicted by the ball upon the anterior crural nerve or its branches. From the 1st of March up to the time of amputation, he had occasional epileptic convulsions, sometimes sufficiently violent to threaten life; a few of these convulsions were tetanic. He lost almost completely the use of language. Although he is well educated and remarkably intelligent, he conversed like a child five years old.

March 14, 1854. Assisted by Drs. Trowbridge and Joseph Smith, I amputated the thigh about six inches above the knee. Patient under the influence of chloroform. No tourniquet applied. The flaps were very ample. Tied a large number of arteries. The femoral artery was contracted, and still conveyed, through the supplies furnished by the anastomosing branches, a small current of blood. I tied it also. I was obliged finally to close the wound, and rely upon pressure to arrest the bleeding from a multitude of small arteries.

As after the first operation, so, also, after this, he had delirium and retention of urine.

March 23. I ascertained that no union was occurring between the surfaces of the flaps, and that by their gradual retraction the bone was becoming exposed. Some sloughing.

June 17, 1854. The skin and muscles had retracted until about two inches of the end of the bone was exposed. Altogether, the retraction of the skin was equal to about seven or eight inches. The general health had improved, and the wound was cicatrizing.

Assisted by Dr. Smith, I removed with a saw the projecting bone. We gave him chloroform only in a moderate quantity, and no unpleasant results followed.

From this time, the stump slowly but steadily became closed.

One additional circumstance deserves notice. The left lung became tuberculous, and finally two abscesses opened into the bronchial passages, and were discharged. His cough then ceased, but the lung is completely consolidated.

